# Current approaches to model extracellular electrical neural microstimulation

**DOI:** 10.3389/fncom.2014.00013

**Published:** 2014-02-19

**Authors:** Sébastien Joucla, Alain Glière, Blaise Yvert

**Affiliations:** ^1^Université de Bordeaux, Institut des Neurosciences Cognitives et Intégratives d'Aquitaine, UMR5287Bordeaux, France; ^2^CNRS, Institut des Neurosciences Cognitives et Intégratives d'Aquitaine, UMR5287Bordeaux, France; ^3^CEA, LETIGrenoble, France; ^4^Inserm, Clinatec, U1167Grenoble, France; ^5^CEA, LETI, ClinatecGrenoble, France

**Keywords:** finite element modeling, extracellular focal microstimulation, microelectrode arrays, neural prosthesis, brain implants, ground surface configuration, compartmentalized neuron models, thin-film approximation

## Abstract

Nowadays, high-density microelectrode arrays provide unprecedented possibilities to precisely activate spatially well-controlled central nervous system (CNS) areas. However, this requires optimizing stimulating devices, which in turn requires a good understanding of the effects of microstimulation on cells and tissues. In this context, modeling approaches provide flexible ways to predict the outcome of electrical stimulation in terms of CNS activation. In this paper, we present state-of-the-art modeling methods with sufficient details to allow the reader to rapidly build numerical models of neuronal extracellular microstimulation. These include (1) the computation of the electrical potential field created by the stimulation in the tissue, and (2) the response of a target neuron to this field. Two main approaches are described: First we describe the classical hybrid approach that combines the finite element modeling of the potential field with the calculation of the neuron's response in a cable equation framework (compartmentalized neuron models). Then, we present a “whole finite element” approach allowing the simultaneous calculation of the extracellular and intracellular potentials, by representing the neuronal membrane with a thin-film approximation. This approach was previously introduced in the frame of neural recording, but has never been implemented to determine the effect of extracellular stimulation on the neural response at a sub-compartment level. Here, we show on an example that the latter modeling scheme can reveal important sub-compartment behavior of the neural membrane that cannot be resolved using the hybrid approach. The goal of this paper is also to describe in detail the practical implementation of these methods to allow the reader to easily build new models using standard software packages. These modeling paradigms, depending on the situation, should help build more efficient high-density neural prostheses for CNS rehabilitation.

## Introduction

Electrical stimulation of the central nervous system (CNS) has been used for decades, both for fundamental research—to decipher the organization and dynamics of neuronal circuits—and with clinical perspectives—to alleviate symptoms of neuronal diseases or restore injured functions (Clark et al., 1977; Benabid et al., 1991; Winfree, 2005). Despite its widespread use, the precise effects of electrical stimulation are far from being understood. For instance, the mechanisms of Deep Brain Stimulation, a popular technique used to treat symptoms of Parkinson's disease and other CNS disabilities, are still debated (Deniau et al., 2010; Shah and Schiff, 2010). The very nature of electrical stimulation—an electrical field flowing through entire structures of the brain—makes it difficult to comprehend its mechanisms and spatial extents using experimental approaches.

To overcome experimental limitations, modeling approaches have been used since the 80's to describe and predict the effects of electrical stimulation on neural elements (McNeal, 1976; Rattay, 1986; Altman and Plonsey, 1990; Rubinstein, 1991) and estimate the activation thresholds of particular electrode configurations (Warman et al., 1992; Holsheimer and Wesselink, 1997; Lertmanorat and Durand, 2004; Rattay and Resatz, 2007; Joucla and Yvert, 2009a; Joucla et al., 2012b). These models are made of two stages: First, the computation of the electrical field created in the tissue by the stimulation; second, the calculation of the response of the target cells to this field. In this article, we will describe and compare two methodologies to address these two calculation steps.

The computation of stimulating electric fields can be performed using a finite element model (FEM). This allows taking into account realistic brain morphologies and electrical parameters (conductivity, dielectric permittivity) (McIntyre and Grill, 2002; Bossetti et al., 2008; Grant and Lowery, 2010; Joucla et al., 2012a; Wongsarnpigoon and Grill, 2012), as well as simple or complex electrode configurations and/or geometries (Rattay and Resatz, 2004; Butson and McIntyre, 2006; Grant and Lowery, 2009; Joucla et al., 2012b). Surprisingly, the properties of stimulation and ground electrodes, and especially their impedance, are generally left aside. In a recent study, we showed however that these properties are essential to simulate electrical stimulation in a realistic manner near the electrode surfaces, in particular in the vicinity of large electrodes, such as the ground electrode (Joucla and Yvert, 2009a).

**Figure 1:**
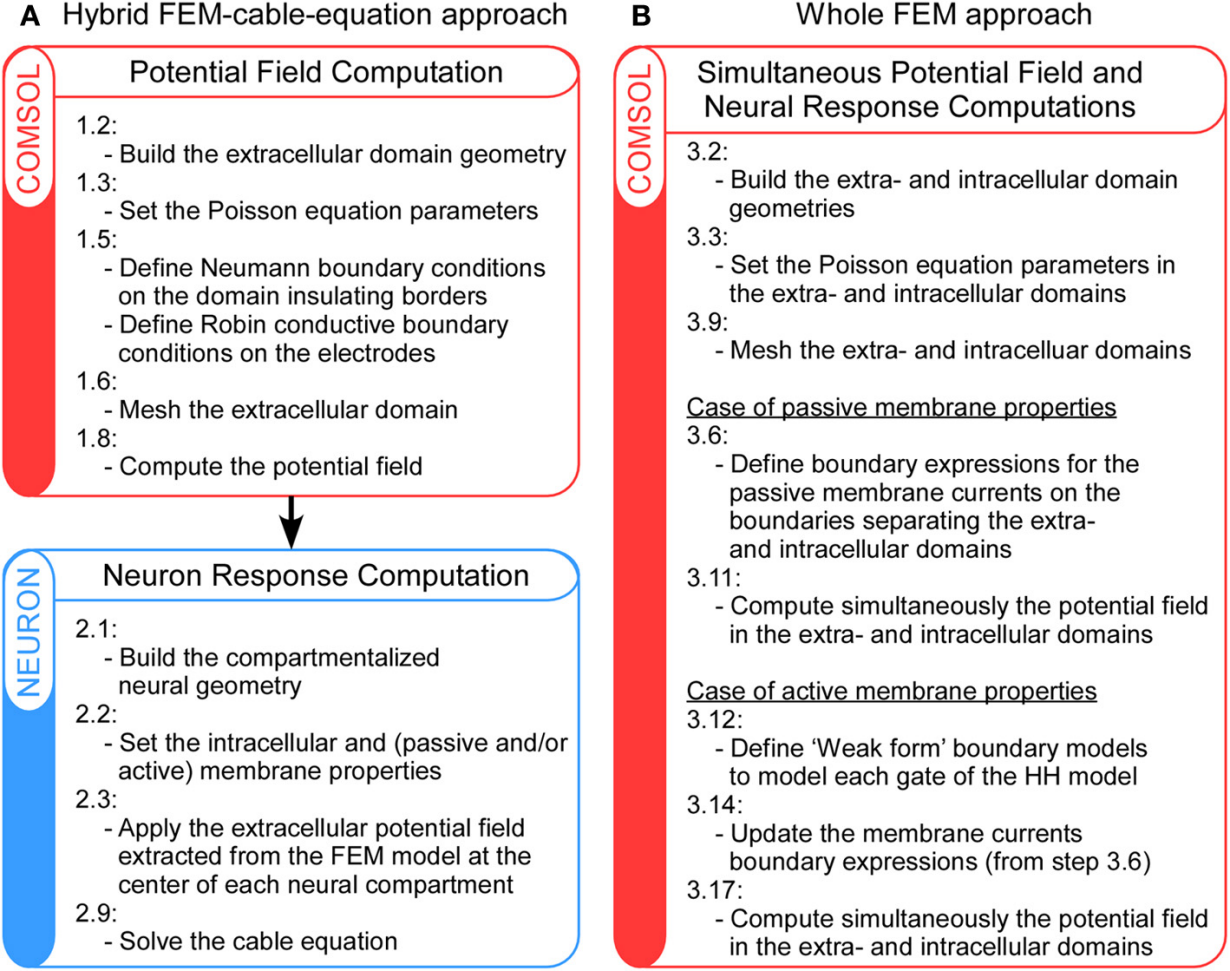
**Schematic representation of the main steps of the hybrid FEM-cable-equation approach (A) and the whole Finite Element Model (B), for the computation of neural response to extracellular electrical stimulation**. For details, refer to the Methods section.

Regarding the calculation of the neuron response, classical approaches generally perform in a separate framework (Greenberg et al., 1999; Miocinovic et al., 2006; Bellinger et al., 2010; Bourbeau et al., 2011), where the target neuron is segmented in compartments and the stimulation potential field—separately calculated with the FEM—is applied at each compartment location. For this purpose, the NEURON software (Hines and Carnevale, 1997) provides a very suitable environment to perform these cable-equation-based calculations. In the following, this will be referred to as the hybrid FEM-cable-equation approach (see Figure 1A).

The above procedure, where the neuron response computation is performed after that of the extracellular potential field, suits for most configurations, but relies on two approximations: (1) The membrane and extracellular potential are uniform over each compartment (in fact, the neurons thickness is not modeled in this framework); and (2) The extracellular potential field is not affected by the presence of the neuron (in fact, the potential field is calculated in a separate model that does not include any microscopic description of the tissue). However, it has been argued that these two assumptions are not valid when target neurons are close to the stimulation electrode (Schnabel and Struijk, 2001). Thus, in such cases, other approaches are required to calculate the neural response to the stimulation. In a recent study (Moulin et al., 2008), we have developed a 3D FEM based on a thin-film approximation, which allows the calculation of the neuron response in the same framework as that used for the computation of the extracellular potential field. Here, we extend this approach to the case of neural stimulation and show that it can reveal important membrane polarization at a sub-compartment level. In the following, this will be referred to as the whole FEM approach (see Figure 1B).

This article is organized as follows: In the first part of the Methods section, we show how to build a FEM to compute the potential field using the software Comsol Multiphysics (COMSOL AB, Stockholm, Sweden) and put special emphasis on the description of the electrical properties of the stimulation and ground electrodes. In the second part, we explain how to implement both the hybrid and whole FEM approaches, using NEURON and the Comsol environment, respectively. In the Results section, we illustrate the usefulness of both approaches for the computation of neural response to extracellular stimuli. In particular, we show that the hybrid FEM-cable-equation approximation can be used in the case of thin fibers and/or dendritic structures, while the whole FEM model allows detailing the precise response of the different parts of the neural membrane in thick regions of the cell such as the cell body. The code corresponding to this example is made available as a supplementary download for both the hybrid and the whole-FEM approaches.

## Methods and protocols

In this article, we present and compare two procedures to model neuronal response to extracellular fields: First, the hybrid FEM-cable-equation approach (Figure 1A), which requires successively the computation of the electrical field [see Hybrid approach step 1: Computation of the extracellular potential field using a Finite Element Model (FEM) below] and the calculation of the neuron response to this field (Hybrid approach step 2: Calculation of the neuron response using NEURON below); Second, the whole FEM approach (Figure 1B), in which both the extracellular and the membrane potentials are computed simultaneously (section Whole FEM approach: Simultaneous calculation of the stimulation potential field and the neuron response below). Procedures to compute the response of both passive and active neurons are presented. User variables and functions in Comsol and Neuron are highlighted in courier font.

**Figure 2:**
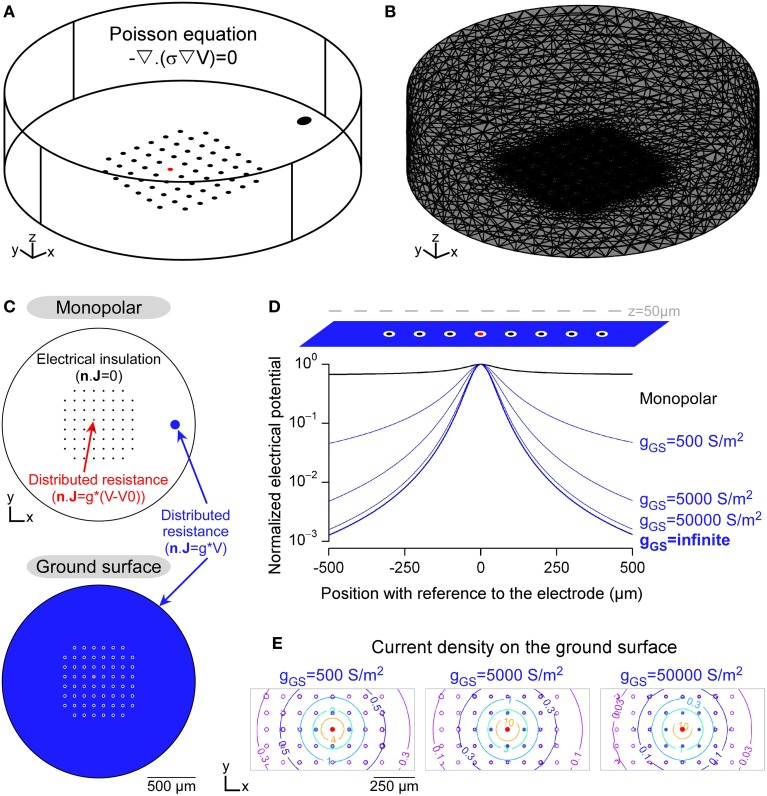
**Modeling the potential field in a FEM including Robin boundary conditions. (A)** 3D geometry of an *in vitro* MultiElectrode Array (MEA) chamber created in the Comsol environment. The Poisson equation was solved in this domain, whose electrical conductivity was 0.2 S/m. **(B)** Mesh of the 3D domain, refined around the electrodes. **(C)** Boundary conditions (BCs) used to model the insulating elements, such as the non-stimulating electrodes and the external limits of the chamber (homogeneous Neumann BC) and the conductive electrodes (Robin BC, modeled in the DC mode of Comsol Multiphysics by a distributed resistance). **(D)** Profiles of electrical potential fields obtained with the monopolar (black) and ground surface (blue) configurations. In the latter case, the influence of the surface conductance of the ground surface was evaluated (values are given in S/m^2^). Electrical potentials, calculated along a line passing 50 μm above the stimulation electrode, were normalized by the maximal value, for each configuration. **(E)** Contour plots of the current density over the ground surface (values are given in A/m^2^).

**Figure 3:**
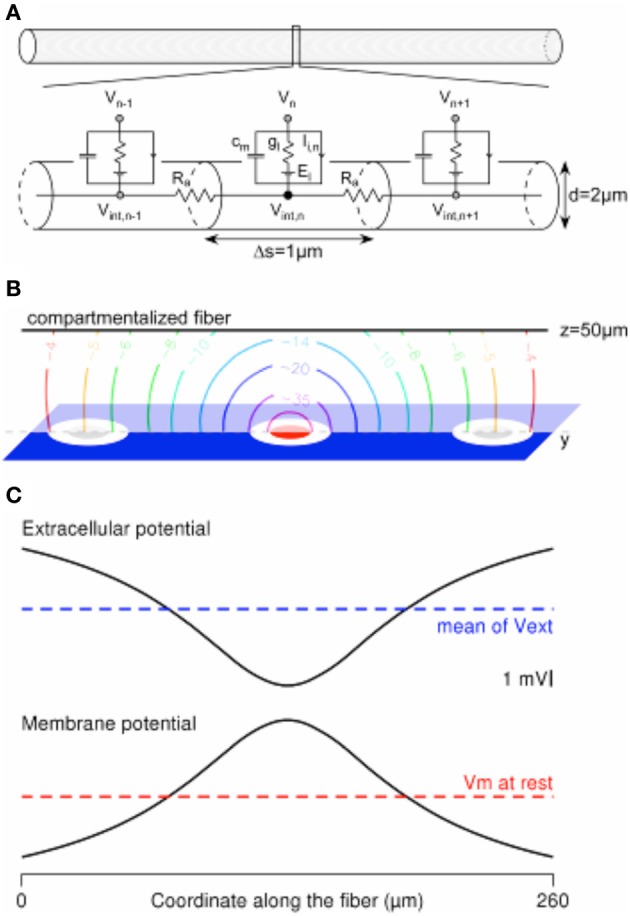
**Computing the mirror response of a uniform passive fiber to an extracellular potential field in the Neuron environment. (A)** A 260-μm-long uniform fiber is modeled in the Neuron environment by a set of electrical compartments linked in series through the intracellular resistance Ra. Each (1-μm-long) compartment is modeled by a capacitance (c_*m*_) in parallel with a membrane conductance (g_l_, in series with a voltage source equal to the leakage potential E_l_), in parallel with ion-specific active currents I_*i,n*_. The latter are set to 0 in the current passive model. **(B)** Contour plot of the extracellular potential field in the y–z plane containing the compartmentalized fiber (oriented along the y axis, at *z* = 50 μm). Values are given in mV, for a cathodic current of −1 μA delivered by the stimulation electrode (in red) and returning through the ground surface (in blue). **(C)** The extracellular potential is plotted at the locations of the fiber compartments, together with the membrane response at the end of a cathodic 1-ms-long stimulation.

**Figure 4:**
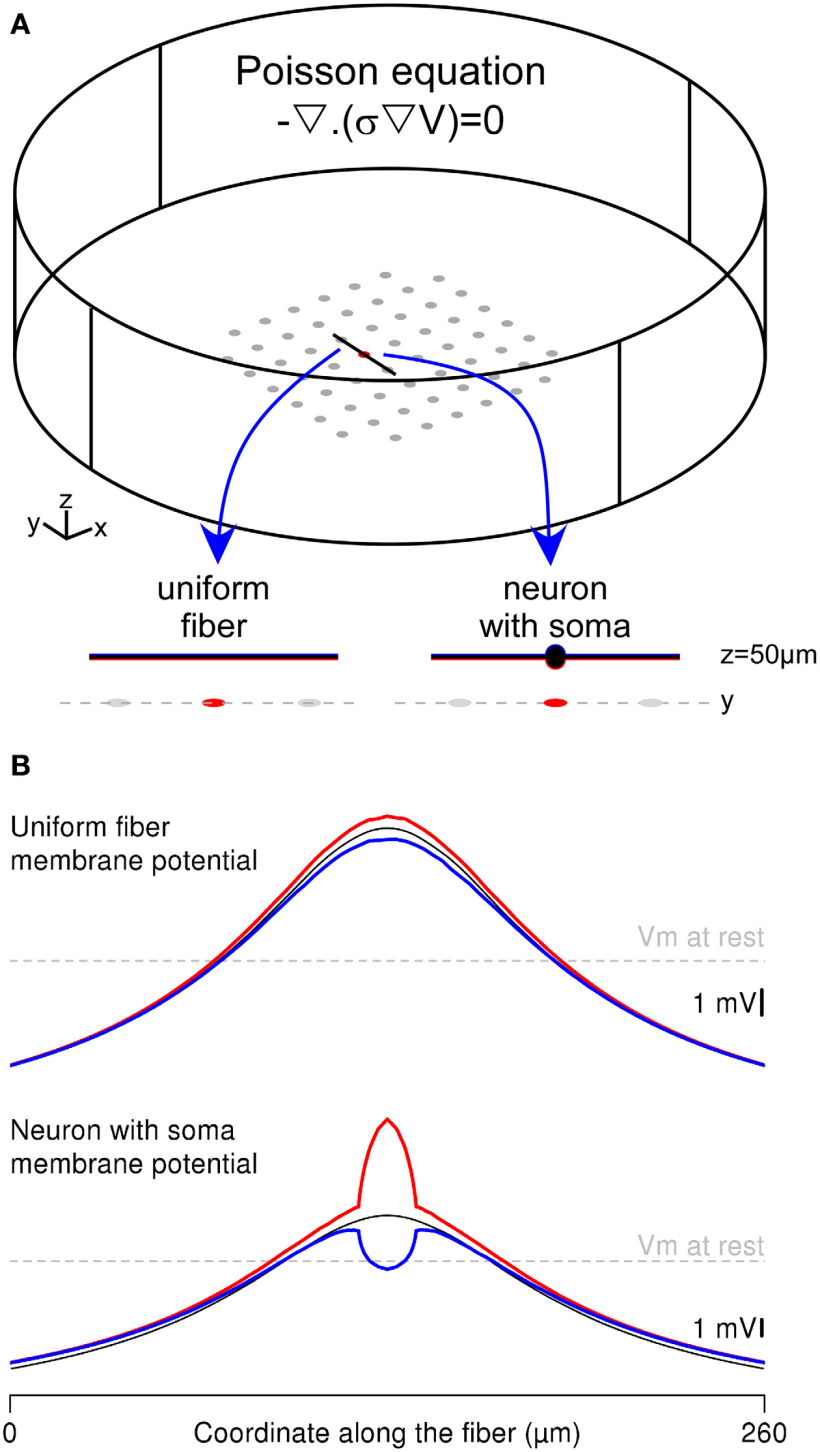
**Complete FEM calculation of the extracellular potential and the neuron response in the Comsol Multiphysics environment. (A)** Two thin-film-approximation-based FEMs were built, representing either a straight uniform fiber or a neuron with a 20-μm-diameter soma in the stimulation chamber. The Poisson equation is solved simultaneously in each domain, with appropriate conductivity values (σ = 0.2 S/m in the extracellular domain and σ = 1 S/m in the neuronal elements). The electrical potentials in the two domains (V and V_int_) are linked through the equations driving the membrane voltage (V_*m*_). The neurons are oriented along the y axis and located above the stimulation electrode at *z* = 50 μm. **(B)** Spatial profile of the membrane potential, plotted along the fiber (top) and neuron (bottom) geometries: In each configuration, the red (respectively blue) curve corresponds to the membrane potential computed in the FEM, along the bottom (respectively top) side of the membrane, facing (respectively, opposite to) the stimulation electrode. The black line corresponds to the solution obtained with the hybrid method.

The primary goal of this paper is to detail the general methods to model extracellular electrical neural stimulation, so that they can be readily used by others. We thus focus on the general presentation of these procedures in the Methods section and then illustrate these approaches on a particular example in the Results section, where the stimulation configuration of a neural tissue (representing either a slice or a whole structure of the Central Nervous System) laid on a 2D microelectrode array (MEA) is detailed. We show how these modeling approaches allow to design a particular electrode configuration aiming at focalizing the potential field created in the tissue (Figure 2) and to model the response of a straight fiber or a neuron taking or not into account the presence of the neural geometry in the calculation of the field (Figures 3, 4).

### Hybrid approach step 1: computation of the extracellular potential field using a finite element model (FEM)

The following procedure describes the different steps to build a realistic finite element model for the calculation of the potential field generated by a current-controlled stimulation. The only requirement is the software Comsol Multiphysics (version 3.5). This procedure computes the potential field generated in the absence of the neuron. This field is thus supposed to be identical if the neuron were present. The different steps are the followings:

1.1 Run the Comsol Multiphysics software. From the “Model Navigator” window, select a 3D model and, in the proposed list, choose Electromagnetics > Conductive Media DC.
1.2 Build the domain geometry, by combining the predefined elementary shapes (e.g., blocks, cones, cylinders, ellipsoids, spheres). Remember that the SI dimension unit is the meter, and create the different shapes accordingly.
1.3 Define the subdomain settings for each volume. In the Conductive Media DC mode, the equation to solve is the following Poisson equation:
  (1) $$-\nabla \cdot (\sigma \nabla \text{V}-J^{e})=-{\text{Q}}_{j},$$
  where σ is the electrical conductivity of the medium (which should be set to a value measured with a conductimeter or taken from the literature, in S/m), **J**^*e*^ is the external current density vector, which should be set to (0, 0, 0) A/m^2^ and Q_*j*_ is the current source (0 A/m^3^).
1.4 Define the element type depending on the expected precision and smoothness of the solution to the problem, and taking into account computation time and computer memory issues. For the Poisson equation (Equation 1), select quadratic Lagrange elements, which approximate the solution by piecewise second degree polynomial functions.
1.5 Define the boundary settings on each surface boundary: These can be of different types, depending on whether the selected element is insulating or conductive.
  1.5.1 For insulating elements, define a homogeneous Neumann boundary condition (BC):
    (2) σ∇V·n=0,
    where ***n*** is the outer vector normal to the boundary. In a Conductive Media DC model, this is done by choosing “Electrical insulation” (***n*** · **J** = 0).
  1.5.2 For each conductive element, define a Robin boundary condition (see Joucla and Yvert, 2009a, 2012 for explanation):
    (3) $$\sigma \nabla \text{V}\cdot n+g\text{V}=g{\text{V}}_{0},$$
    where V_0_ is the voltage taken by the electrode on the metal side (not on the medium side) and g is the surface conductance of the electrode-medium interface. In a Conductive Media DC model, this is done by choosing “Distributed resistance”: In this mode, the Robin BC is written as ***n*** · **J** = σ(V - V_ref_)/d, where d is related to the thickness of the electrode-medium interface. To fully define such BC, proceed this way:
    – Set σ to the value defined in the subdomain settings.
    – Compute the surface conductance g of the interface as the inverse of the electrode impedance (Z_elec_) divided by the electrode area (S_elec_). g should be in S/m^2^.
    – Set d to σ / g.
    – Set V_ref_ to 0 for the ground electrode.
    – Set V_ref_ to V0 for the stimulation electrode(s). V0 should be computed so as to set the desired current *I*_elec_ actually flowing through the electrode. For that purpose, set V0 to 1 Volt in a first approach and compute the total current *I* flowing through the stimulation electrode, at the end of the simulation (see below step 1.9). Then, in a second time, modify V0 so that the expected *I* is equal to *I*_elec_, and solve the problem a second time. In practical cases where the electrode impedance is high, the voltage V in the medium in front of the electrode is small compared to V0 and the total current flowing through the electrode boundary can be approximated by *I*_elec_ = σ V0/d S_elec_ (*I*_elec_ is in A). Set V0 accordingly (in V).
1.6 Define a (tetrahedral) mesh size in the whole domain. Refine it locally in the regions where more precise calculations are required (with a typical element edge size of less than 5 μm). This is for instance the case around the locations where the neuron used in the second step of the protocol (using NEURON) will be located, or also on and around the stimulation electrode. In the latter case, an accurate calculation of V will lead to an accurate estimation of the actual current flowing through the electrode.
1.7 Select the parameters of the Stationary solver. If the number of degrees of freedom (DoFs) is small enough, a direct resolution of the meshed problem can be performed, for instance by choosing the Direct (UMFPACK) solver. The threshold number of DoFs depends on the computer characteristics, but typically, this works for a mesh with less than 100,000–150,000 DoFs. For denser meshes, iterative algorithms can be used, such as the conjugate gradient algorithm preconditioned with the SSOR method.
1.8 Solve the problem.
1.9 Check that the total current flowing into the tissue equals the desired nominal current. For that purpose, integrate the norm of the current density over the boundary of the stimulation electrode (this gives *I*_1_). Do it also on the boundary of the ground electrode (this gives *I*_2_). Computing the current density requires calculating the derivatives of V and is thus subject to errors. For this reason, the two currents calculated previously (*I*_1_ and *I*_2_), which should be opposite one from the other, may differ significantly. In such case, trust the current calculated on the larger electrode, since it is generally computed on a greater number of surface triangles.
1.10 If the calculated total current does not equal the nominal one, multiply V0 by their ratio (step 1.5.2) and run the problem again).

Having completed the previous steps, interpolate the values of the electrical potential V at the locations of the centers of the neuron compartments. For that purpose, use a cross-section plot (from the postprocessing menu) and save the results in a.txt file.

### Hybrid approach step 2: calculation of the neuron response using neuron

The following procedure describes how to simulate the response of a neuron to an extracellular potential field previously calculated and interpolated at the center of each compartment. The model is created in the free software NEURON (version 7.2 or later) and requires only the knowledge of the distribution of the extracellular potential along the neuron, saved previously in a text file for instance.

For this procedure, we do not use the NEURON's graphical interface, but only a.hoc script gathering all the following instructions.

2.1 Create the neuron morphology in 3D. For that purpose, create each compartment using the pt3dadd function so as to keep the 3 dimensional organization of the neuron. Use small-enough compartments to get accurate computation. In practice use a compartment size so that using a twice smaller one would lead to < 1% difference in the membrane potential V_*m*_ (usually of the order of 1 μm for a 100-μm-long axon). Connect the created compartments in the correct order, using the connect function recursively.
2.2 Define the electrical properties of the neuron: For each compartment, set the intracellular resistivity Ra (in Ohm.cm), the surface capacitance cm (in μF/cm^2^) and the active and passive conductances. This can be done using the Hodgkin and Huxley hh mechanism (Hodgkin and Huxley, 1952) or user-defined membrane currents. In the first case, for instance, define the membrane leakage conductance gl_hh (in S/cm^2^) and the sodium and potassium maximal conductances gnabar_hh and gkbar_hh in the compartments where the associated channels are located. Define also the leakage potential El as well as the potassium and sodium equilibrium potentials (EK and ENa). Proceed identically for user-defined conductances.
2.3 To model electrical stimulation, insert the extracellular mechanism in all compartments. This creates a variable e_extracellular in each compartment. This variable takes a different value at each compartment and at each time step during the stimulation pulse.
2.4 Define the integration scheme by setting the variable secondorder to 0. Doing so, you select the first-order backward Euler scheme. If you set secondorder to 1, you will use the second-order Crank-Nicholson scheme, but this does not work properly with the extracellular mechanism (Carnevale and Hines, 2006).
2.5 Set the integration time step by assigning the variable dt the desired value. This value should be chosen so as to have a sufficient number of time samples during the stimulation pulse and the neuron action potential (if present). So, a typical value for dt is 0.05 ms or less. A very small value for dt will give very accurate results, but at the price of an increased computation time. If an important set of simulations has to be run, it can be good to evaluate the accuracy of the solution for various time steps and choose a value of dt that gives a good compromise between accuracy and computation time. To determine a good value of dt, compute the solution of the cable equation for dt and dt/10 and test whether the relative difference of the membrane potential (V_*m*_) at a given position (for instance, the location of the largest value of V_*m*_) is smaller than 1%. If this is not the case, divide dt by 10 until this criterion is satisfied.
2.6 Define a piecewise or pointwise stimulation time course. This is the function by which the extracellular potential will be multiplied over time, for each compartment. This can be done by defining a different value for different time windows, when using rectangular functions (for instance −1 for a cathodal pulse,+1 for an anodal pulse, 0 before and after the stimulation, or even between the different pulses). You can also create a vector storing the amplitude of the stimulation time course for each time step, which can be useful if other functions (more complex than rectangles) are needed.
2.7 Set the initial value of the membrane potential using the finitialize function. This value can be that of the resting potential.
2.8 Initialize the simulation using the init function.
2.9 Until the end of the simulation has not been reached, update the current value of the extracellular potential (e_extracellular) at each compartment and integrate the equation over one time step by calling the fadvance function. Note that updating the value of the extracellular potential at each time step allows defining complex stimulation temporal patterns, such as succession of rectangle functions of different amplitudes, or sinusoids, and so on. This is the reason why the stimulation time course should then be defined pointwise or piecewise before running the simulation (stage 2.6 above).
  Modify steps 2.6–2.8 depending on the objective of the study. For instance, if your aim is to determine the threshold intensity at which the target neuron fires an action potential, embed these steps in a dichotomy algorithm in which the global amplitude of the stimulation time course (step 2.6) will be increased or decreased until threshold is reached. At each step of this dichotomy, the action potential is detected when the membrane potential of a given compartment (or a set of compartments) exceeds the spike threshold.

### Whole FEM approach: simultaneous calculation of the stimulation potential field and the neuron response

As stated in the Introduction, another approach to model extracellular stimulation is to use the FEM in the Comsol environment as in section Hybrid approach step 1: Computation of the extracellular potential field using a Finite Element Model (FEM), and also embed the geometry of the neuron in this model to compute its response. For this purpose, the following procedure can be used.

3.1. Run the Comsol Multiphysics software and open the Finite Element Model created in section Hybrid approach step 1: Computation of the extracellular potential field using a Finite Element Model (FEM). From the “Model Navigator” window, add a new Conductive Media DC model, the variable of which is named Vint.
3.2. Build the geometry corresponding to the target neuron. This separates the whole geometry in two domains: The intracellular domain corresponding to the neuron (noted domain #2 in the following) and the extracellular domain (noted domain #1 in the following). Domain #1 corresponds to the original domain from which the neuron has been removed. However, note that this separation is somewhat “virtual” and not definitive, so that the geometrical characteristics of the neuron can be easily modified, without having to build again domains #1 and #2.
3.3. From the multiphysics section, select the 1st DC model. Set the geometry #1 active and the geometry #2 inactive in this domain. Then, select the 2nd DC model. Set the geometry #2 active and the geometry #1 inactive in this domain. In each case, define the value of σ. For the neuron geometry, σ is equal to the inverse of Ra expressed in Ohm.m.
3.4. For each geometry, define the initial value (at *t* = 0) of the variable. In domain #1, set V–0 (the default value). In domain #2, set Vint to the neuron resting potential (this corresponds to step 2.7 in the Neuron simulation).
3.5. Define the element type depending on the expected precision and smoothness of the solution to the problem, and taking into account computation time and computer memory issues. In the current simulation paradigm, the extracellular potential is not interpolated so as to be used in another software (such as NEURON), but the extracellular and intracellular potentials (V and Vint) are computed simultaneously. For sake of computational efficiency, use linear Lagrange elements in domain #1 and quadratic Lagrange elements in the neuron geometry (domain #2). This gives accurate results without increasing too much the computation time.
3.6. Define the boundary settings on the boundaries between the neuron and the extracellular domain. In the present case, we first consider the case of a passive neuron not equipped with voltage-dependent conductances. The case of an active neuron is provided further below.
  3.6.1. Select the 1st DC model and assess an “Inward Current Flow” boundary condition to the boundaries between the extracellular domain and the neuron. This BC is written −***n*** · **J** = J_n_. Set J_n_ to Im, which represents the total current flowing through the neuron membrane. Im is given in A/m^2^. Define Im as a “Boundary expression” for convenience (see below).
  3.6.2. Select the 2nd DC model and assess an “Inward Current Flow” boundary condition to the boundaries between the neuron and the extracellular domain. Set J_*n*_ to –Im.
  3.6.3. Define a boundary expression for Im. In the passive case, the neuron membrane is represented by a capacitance in parallel with a conductance (which is in series with a voltage source, see Figure 3A). Thus, the membrane current is the sum of the capacitive and resistive leakage currents. Define a boundary expression for each of these currents and for the total membrane current:

- Ic = cm*(Vintt-Vt),
- Il = gl*(Vint-V-El),
- Im = Ic + Il,

where Vintt and Vt represent the first-order time derivatives of Vint and V, respectively, and El is the neuron leakage potential.
3.7. Define a time-dependent boundary condition on the stimulation electrode(s), corresponding to the desired stimulation time course [noted f(t)]. For that purpose, modify the value of V_ref_ in the “Distributed resistance” BC, which was initially set to V0. Set it to V0*f(t). Define f as a function of argument t using the dedicated “Functions” tool. For instance, define cathodic and anodic rectangle stimuli with a piecewise polynomial function.
3.8. Define constant values for the electrical properties of the neuron: cm, gl and El.
3.9. Define a mesh size in domains #1 and #2, and refine it locally on the neuron boundaries. This is important, especially if you use linear Lagrange elements in geometry #1.
3.10. Select the parameters of the Time-dependent solvers. Use the Direct (UMFPACK) method as linear system solver and set the time steps in accordance with the time constant of the problem. Give small values to the absolute and relative tolerances, taking into account the resulting computation time.
3.11. Solve the problem.
  The case of a passive neuron was presented first since it allows the explanation of specific characteristics of the current model, compared to the original one: simultaneous resolution of two equations (this takes advantage of the Multiphysics approach of Comsol) linked by boundary conditions (BCs) at the neuron membrane, as well as time-dependence of the problem to solve. In practice, neurons contain active (voltage-dependent) conductances, which must be taken into account when calculating the total membrane current Im. Here, we show how to model Hodgkin-Huxley-like voltage-dependent conductances in the Comsol environment.
  The equations driving the potassium and sodium membrane currents are the following, as given by Hodgkin and Huxley (1952):
  (4a) $$I_{K}=g_{K}\cdot \left(V_{m}-E_{K}\right)$$
  (4b) $$\text{and }I_{Na}=g_{Na}\cdot \left(V_{m}-E_{Na}\right)\text{​},$$
  where E_*K*_ and E_*Na*_ are the equilibrium potentials of the potassium and sodium ions, and g_*K*_ and g_*Na*_ are voltage-dependent conductances. The latter can be expressed as:
  (5a) $$g_{K}=\bar{g_{K}}\cdot n^{4}$$
  (5b) $$\text{and }g_{Na}=\bar{g_{Na}}\cdot m^{3}\cdot h,$$
  where *n*, *m*, and *h* represents the opening probabilities of the *n*-, *m*- and *h*-gates constituting the channels. *n*, *m* and *h* can be calculated from the following first-order differential equations:
  (6a) $$\frac{dn}{dt}=\alpha_{n}\cdot \left(\text{1}-n\right)-\beta_{n}\cdot n,$$
  (6b) $$\frac{dm}{dt}=\alpha_{m}\cdot \left(\text{1}-m\right)-\beta_{m}\cdot m$$
  (6c) $$\frac{dh}{dt}=\alpha_{h}\cdot \left(\text{1}-h\right)-\beta_{h}\cdot h,$$
  where:
  (7a) $$\alpha_{n}=\frac{\text{0}.\text{01}\cdot \left({\tilde{V}}_{m}+\text{10}\right)}{\text{exp​}\left(\left({\tilde{V}}_{m}+\text{10}\right)\text{​}/\text{10}\right)-\text{1}}\text{ and }\beta_{n}=\text{0}.\text{125​}\cdot \text{​exp}\left({\tilde{V}}_{m}/\text{80}\right)\text{​},\;$$
  (7b) $$\alpha_{m}=\frac{\text{0}.\text{1}\cdot \left({\tilde{V}}_{m}+\text{25}\right)}{\text{exp​}\left(\left({\tilde{V}}_{m}+\text{25}\right)\text{​}/\text{10}\right)-\text{1}}\text{ and }\beta_{m}=\text{4​}\cdot \text{​exp​}\left({\tilde{V}}_{m}/\text{18}\right)\text{​},$$
  (7c) $$\alpha_{h}=\text{0}.\text{07}\cdot \text{exp​}\left({\tilde{V}}_{m}/\text{20}\right)\text{ and }\beta_{h}=\frac{1}{\text{exp​}\left(\left({\tilde{V}}_{m}+\text{30}\right)\text{​}/\text{10}\right)+\text{1}}.$$
  In these expressions, ${\tilde{V}}_{m}$ denotes the opposite of the variation of the membrane potential around its resting value.
  To simulate these equations in Comsol, use the following procedure:
3.12. From the “Model Navigator” window, add 3 “Weak form” boundary models, the variables of which are named N, M, and H. This defines 3 variables which take values only on the boundaries of the domain.
3.13. From the multiphysics section, select 1 weak form, boundary, for instance N. Set this model active on the boundaries of the neuron geometry in which potassium channels are present. Set the model inactive on all other boundaries. Do the same for the M and H variables, by selecting the boundaries where sodium channels are present.
3.14. Set the boundary settings of variable N as follows (do the same for variables M and H). On the boundaries on which N is active:
  3.14.1. Set weak' to N_test * (alpha_N - (alpha_N + beta_N) * N).
  3.14.2. Set 'dweak' to N_test * Nt. These two steps correspond to Equations (6) above, multiplied by a test function N_test on both sides, accordingly with the weak form approach.
  3.14.3. Set the initial value of n: N(t0) to N0. The value of N0 is calculated from Equations (6) when the membrane potential is at its resting value, i.e., when dN/dt = 0. Then, set N0 to alpha_N0 / (alpha_N0+beta_N0).
  3.14.4. Set the initial value of the time-derivative of *n* to 0 (Nt(t0)=0).
3.15. In the boundary expressions, define new expressions for alpha_N, beta_N, alpha_M, beta_M, alpha_H and beta_H, from Equations (7). Define also the voltage-dependent conductances gK and gNa and eventually the active currents IK and INa. Update also the expression of Im on the relevant boundaries:

- IK = gK* (Vint-V-EK),
- INa = gNa* (Vint-V-ENa),
- Im = Ic + Il + IK + INa.

3.16. Define constant values for gK_bar, EK, alpha_N0, beta_N0, gNa_bar, ENa, alpha_M0, beta_M0, alpha_H0 and beta_H0.
3.17. Solve the problem.

## Results

In this section, we illustrate and compare the use of the hybrid FEM-cable-equation and whole FEM approaches. Regarding the calculation of the potential field, we focus on the ground surface configuration, a case where the choice of the BCs for stimulation and ground electrodes is crucial [section Hybrid approach step 1: Computation of the extracellular potential field using a Finite Element Model (FEM)]. The potential field generated in the tissue is then applied to a straight fiber (section Hybrid approach step 2: Calculation of the neuron response using NEURON) and a neuron with a cell body. In a previous study, we showed that the neural response follows the mirror image of the extracellular potential field along the neuron. Here, we compare this mirror response in both approaches. We found that the whole-FEM approach, which takes into account the neuron morphology in a whole finite element model, allows to see a clear difference of the neuron response between the sides the soma membrane either facing or opposite to the stimulating electrode (section Whole FEM approach: Simultaneous calculation of the stimulation potential field and the neuron response).

### Importance of the robin boundary condition to model the potential field

Figure 2 shows a finite element model used to compute the electrical potential field created by a stimulation in a neural tissue. The stimulations were applied in a cylindrical chamber surrounding a 8-by-8 MultiElectrode Array (MEA) without the corners comprising 60 10-μm-diameter planar microelectrodes spaced every 100 μm, similar to standard designs used in the community for *in vitro* applications (Figure 2A). The potential field was computed by solving the Poisson equation (Equation 1) in which the electrical conductivity was set to 0.2 S/m, a value which falls in the range of reported neural tissues electrical conductivities (0.1–0.4 S/m Ranck et al., 1963, 1965; Geddes and Baker, 1967). For sake of simplicity, we modeled a single domain with uniform conductivity, assuming that the tissue filled the whole chamber. As reported previously (Joucla and Yvert, 2009a; Joucla et al., 2012b), more complex tissues with several domains of various conductivities can be modeled by adding current continuity BCs at the domain interfaces. The 3D domain was meshed with about 100,000 tetrahedral elements and locally refined on the substrate of the MEA (Figure 2B). The focality of the potential field created by two electrode configurations were compared: First, the classical monopolar configuration, in which the stimulation is applied between a stimulation electrode and a distant ground electrode; Second, the recently proposed “ground surface” configuration, which consists in adding a layer of conductive material on the MEA chamber substrate and using this surface as ground electrode (Figure 2C). The gap between each electrode and the ground surface was set to 10 μm.

Figure 2D illustrates the profile of the potential field along a line passing 50 μm above the stimulation electrode, and shows that the potential field created by the ground surface configuration was more focal than that created by the monopolar configuration. Moreover, the field focality increased when the surface conductance (g) of the ground surface increased, starting from g_GS_ = 500 S/m^2^, a typical value for Platinum electrodes (Figure 2D). We emphasize that this influence of the surface conductance has been obtained with a model including the Robin boundary condition (BC), which directly depends on g. With the homogeneous Dirichlet boundary condition, which imposes a zero potential on the ground electrode (*V* = 0), the potential field would have been that obtained with an infinite surface conductance (g_infinite_ in Figure 2D), and thus would not have reflected the actual shape of the extracellular potential. Moreover, as shown in Figure 2E, the current density is not uniform over the ground surface. Thus, the Robin BC could also not be replaced with a non-homogeneous Neumann BC imposing a uniform current density (***n*** · **J =** i).

### The mirror response of a fiber with the hybrid approach

Figure 3 shows a model of a compartmentalized fiber stimulated by an extracellular potential field. The computations were done in the Neuron environment. We considered a 2-μm-diameter-large and 260-μm-long passive uniform fiber (Figure 3A), which was segmented in 1-μm-long compartments to ensure accurate computation of its response to the field (Joucla and Yvert, 2009b). The electrical parameters were set to the following values: *Ra* = 100 Ohm.cm, c_*m*_ = 1 μF/cm^2^, *g_l_* = 10^−4^ mS/cm^2^, *E_l_* = − 65 mV, while the ionic currents (*I_i_*) were set to 0. The electrical potential field created in the neural tissue by a −1 μA cathodic ground surface stimulation (Figure 3B, modeled in the Comsol Multiphysics software, with g_GS_ = 500 S/m^2^) was interpolated at the locations of the fiber compartments, passing above the stimulation electrode at *z* = 50 μm (Figure 3C-top). As shown in Figure 3C-bottom, the membrane potential profile (centered on the resting membrane potential) computed at the end of the 1ms-long stimulation is the mirror image of the extracellular potential centered on its spatial average along the fiber geometry (Joucla and Yvert, 2009b).

### The mirror response of a fiber and a neuron with the whole-FEM approach

An alternative to the hybrid approach consists in embedding the neuron geometry in the finite element model and computing simultaneously the extracellular potential field and the neuron intracellular potential with a thin-film approach. We implemented this approach in Comsol Multiphysics to compute the membrane response of both the uniform passive fiber modeled in Neuron and a neuron of identical length including a 20-μm-diameter cell body (Figure 4A). These two neuronal geometries were stimulated with a ground surface configuration (*g*_GS_ = 500 S/m^2^). We focused on the spatial distribution of the membrane response along 2 sides of the neuron: The bottom side facing the stimulation electrode (in red) and the top side opposite to the electrode (in blue), and compared these with the response obtained in the Neuron environment (thin black curves).

As shown in Figure 4B-top, the fiber response obtained with the thin film model was identical to the cable response at the ends of the fiber, and slightly differed in the middle of the fiber (compare the blue and red curves with the black one). This was due to the potential field gradient across the fiber geometry (in the *z* direction), which was most important above the stimulation electrode. Nevertheless, the differences between the top and bottom sides of the membrane were very small, because of the small fiber diameter (2 μm).

By contrast, the neuron-with-soma response greatly differed between the compartmentalized and the thin-film approaches. Indeed, in the full finite element model, membrane potential computed at the level of the soma (above the stimulation electrode) displayed large variations between the bottom and the top sides of the soma (Figure 4B-bottom). This stems from both the large soma diameter and the important potential field gradient across the neuron geometry (in the *z* direction). Contrary to the fiber response, the variations of the membrane response along the soma contor were not symmetrical with respect to the cable response obtained in Neuron. This was due to the decrease of the potential field gradient along the z axis. Indeed, the bottom side of the membrane is located at *z* = 40 μm, where the field gradients are most important than at *z* = 60 μm, which is the location of the top side of the membrane. Thus, the largest membrane potential variations around the resting potential were obtained on the side facing the stimulation electrode (see red curve). This example shows that the whole-FEM approach allows catching important membrane response features at the sub-compartment level, which cannot be tackled using the classical hybrid approach.

## Discussion

This article aims at presenting two different methods to model electrical stimulation of neurons at the single-cell level. Basically, neurons are excited by the potential field created in a conductive neural tissue by the electrical stimulation. The accuracy of the stimulation modeling depends on the relevance of both the model used to compute the electrical potential field and that used to calculate the neuron response. The neuron response can be computed either with a cable equation formalism or by embedding the neuron morphology in a complete FEM model. In the latter case, the proposed approach treats the membrane as a thin film, which has the advantage not to require meshing and specifying explicitly its volume.

Regarding the computation of the electrical potential field, the simplest model is the point source electrode, which applies in infinite media with uniform and homogeneous electrical conductivity (Plonsey, 1969). Although this configuration does not correspond to the case of complex nervous tissues in contact (or implanted) with electrodes having non-negligible sizes, such model has long been used to study the basic mechanisms of neuronal activation with externally applied fields (Rattay, 1989; Rubinstein, 1993; Plonsey and Barr, 1998; Wesselink et al., 1998; McIntyre and Grill, 2000). More realistic modeling takes into account the actual size of the electrodes, which requires numerical approaches. Following the widespread of numerical methods and efficient computers, finite difference methods, based on the approximation of the Poisson equation on a grid, have been developed (Struijk et al., 1992, 1993; Holsheimer and Wesselink, 1997; Wesselink et al., 1998). Since about 15 years, they have been supplanted with finite-element models, which allow an easy resolution of the Poisson equation in domains with complex geometries and electrical parameters (McIntyre and Grill, 2002; Rattay and Resatz, 2004; Butson and McIntyre, 2006; Grant and Lowery, 2009, 2010; Wongsarnpigoon and Grill, 2012; Joucla et al., 2012a,b).

A key element of numerical models is the choice of the BCs, which determine a unique solution to the Poisson equation. Here, we focused on the Robin BC, which has the advantage of taking into account the potential drop at the electrode-tissue interface, through the (non-infinite) surface conductance of the electrodes (Joucla and Yvert, 2009a). We emphasize that the vast majority of literature models use Dirichlet BCs, which consists in setting the electrodes potentials to a uniform value on the electrode surface, thus ignoring the behavior of the interface during stimulation, and assuming this surface as infinitely conductive, which is not the case in practice with typical electrode materials. In the particular example of the ground surface configuration, the Robin BC allowed to highlight the influence of the ground surface conductance on the focalization of the potential field created in the tissue, which would not have been made possible with the Dirichlet BC.

Further applications of this approach may include the design of neural implants for specific applications requiring precise electrical microstimulation of the CNS. The precise modeling of the extracellular potential field could indeed help to build specific electrode configurations designed to specifically target pre-determined group of cells depending on the application (e.g., retinal implants or cortical neural prosthesis). Further improvements could be brought to the present modeling approach to account for the frequency dependence of stimulation waveforms (Bossetti et al., 2008). For instance, in many stimulation paradigms, electrical stimuli are delivered as rectangular functions, during which the (complex) electrode impedance may vary. Thus, an improvement of the model would consist in assigning time-dependent surface conductances to the electrodes, by combining the Fourier transform of the electrical stimuli and the frequency-dependence of the electrodes' impedance.

Once a correct model of the extracellular potential field has been determined, the response of a modeled neuron has to be calculated. For that purpose, a cable equation, relating the membrane potential to the extracellular potential, is classically solved. This cable equation (see Joucla and Yvert, 2009b for instance) is discretized in space using finite differences, leading to a system of *N* linearly dependent first-order differential equations (*N* corresponding to the number of compartments in the neuron geometry). This system of equations can be solved in a home-made simulation program or in a dedicated environment, such as the GENESIS (Bower and Beeman, 1998) or NEURON software packages (Hines and Carnevale, 1997). We used the latter, a tool of choice for subcellular to network simulations (according to the NEURON website, more than 1176 publications have been using NEURON as of January 29, 2012).

NEURON models can be built either graphically or using the associated script language. We used scripts, which allowed an easy implementation of the extracellular stimulation protocol, using the extracellular process. We note that, although the source term of the cable equation is the spatial second derivative of the extracellular potential along the neuron geometry, the extracellular process works by defining only the electrical potential at the location of each compartment, the approximated spatial second derivative being calculated internally by NEURON based on the 3D neuron geometry. This allows a very easy implementation of extracellular stimulation paradigms, since the only mandatory step is to interpolate the extracellular potential at the corresponding coordinates in the FEM model. Regarding practical implementation of an extracellular process, it should be noticed that the cable equation should be time-discretized using the first-order backward Euler scheme and not the second-order Crank-Nicholson scheme, since the latter is prone to oscillations and large errors (Carnevale and Hines, 2006). The backward Euler scheme is chosen by setting the secondorder variable to 0.

Models created in the NEURON environment are based on a 1D cable approach, derived from the initial formulation of McNeal (1976) and Rattay (1986). Such formulation considers that the neuron response varies only in the longitudinal direction and not along the contour of the compartments' membrane. Moreover, it assumes that the presence of the neuron does not affect the extracellular potential field and that this field varies only along the neuron direction and not in the orthogonal directions. However, it has been shown that these assumptions are only fulfilled within an intermediate range of electrode-to-fiber distances, of the order of 100 μm–1 cm (Schnabel and Struijk, 2001). Nowadays, stimulation devices are used not only for peripheral nerve stimulation, but also increasingly for CNS microactivation. In such case, the aim is to specifically activate local pools of neurons involved in specific task or behavior. The distance between the target neurons and the stimulation electrode(s) tends to decrease below the 100-μm limit. Consequently, appropriate simulation tools taking into account the presence of the neuron in the field should be developed in order to predict the actual effects of local stimulation more precisely than with the cable approach.

To this end, a hybrid finite element model has been developed by Ying and Henriquez (2007), which solves simultaneously the Poisson equation in the intracellular and extracellular domains, and models the membrane as an infinitely thin interface. This model uses an iterative algorithm combining a spatial resolution in the FEM and a time integration done separately. However, it remains limited to 2D circular or spherical cells or arrangements of cells placed in uniform electrical fields (Pourtaheri et al., 2009), and therefore does not cover a wide range of practical situations. More recently, an asymptotic model has been developed, that solves the 3D boundary value problem by coupling a 2D “transverse” problem and a 1D “longitudinal” problem (Cranford et al., 2012). This, model, which also treats the fiber membrane as infinitely thin, relies on an asymptotic separation between the fast fiber response (its initial polarization) and its slow response (depending on the membrane time constant), together with a separation between the short and long spatial scales, determined respectively by the fiber radius and length constant. This approach showed promising results for long and thin fibers, but might not be directly extended to the case of complex neuron morphologies including branching points and large diameters at the cell body, since the asymptotic approximation might not be valid in this case.

To overcome these drawbacks, we presented here how to use a thin-film-approximation-based finite element model, which embeds complex neuronal geometries in 3D extracellular fields created by various electrode configurations. This model, implemented in the Comsol Multiphysics environment, was first developed to study the extracellular fields generated in the extracellular medium by spiking neurons and their recording by microelectrodes (Moulin et al., 2008). Similarly to the above-cited models, it simultaneously solves the Poisson equation in two domains that are coupled by a time-dependent boundary condition (BC), the resolution being done in a single environment, without any approximation related to the geometrical and electrical parameters of the modeled cell. A strength of this model is that both passive and active membrane properties can be easily simulated, through a simple modification of the BC describing the membrane currents. Moreover, the cell geometry can include branching points and diameter variations, which allows extending the simulation paradigms to complex CNS neurons.

It should be noted that this thin-film approximation method proposed here is close to the bidomain approach used previously to model intracellular response of cardiac tissue (Roth and Wikswo, 1994) or more recently neural stimulation of the retina (Dokos et al., 2005). In the latter study, the retina was modeled as a whole by a large intracellular volume thus not accounting for the precise morphology of individual neurons. By contrast, here we model directly the 3D geometry of a neuron, in order to detail the response of the whole membrane morphology to an extracellular stimulation.

The whole-FEM approach gave similar results to the compartment NEURON model in the case of a uniform fiber. However when a cell body was introduced (Figure 4B, bottom), it revealed important sub-compartment behaviors of the neural membrane that could not be seen using the classical compartmental approach. More precisely, the cable formalism predicted well the cell response averaged within each compartment, but, within a given compartment, the FEM approach gave more detailed description of the membrane response. For instance, in the present example, the membrane area close to the electrode was more depolarized by a cathodic stimulus than membrane area located on the side opposite to the electrode. Although this effect was almost negligible in the case of a fiber, much larger variations between opposite membrane sides were observed with a neuron geometry including a cell body. For the small electrode-to-neuron distance considered here (50 μm), these transverse-field-induced variations were of the same order as those induced by the longitudinal field, showing the importance of a full 3D model to correctly account for the effects of electrical stimulation at short distances.

A practical aspect for the use of this model is the computation time. The models built for this paper consisted in about 75,000 DoF. Stimulation were applied as 1-ms-long rectangular functions, starting after a delay of 1 ms. The time required for a 20-ms-long simulation was of the order of 400 s for a passive model. This relatively fast computation time, as compared to that reported in Cranford et al. (2012)—about 28 min—was enabled by the use of an adaptive integration time step in the COMSOL solver.

In summary, we presented state-of-the-art models that allow relevant simulation of electrical stimulation of CNS neurons. These models are first based on a correct description of the extracellular field, which should be calculated from a boundary value problem embedding Robin boundary conditions at the stimulation and ground electrodes. This field can then be applied to a compartmentalized neuron model to compute its response in the case of large enough electrode-to-neuron distances. For short distances, the computation of the extracellular potential should be performed simultaneously with that of the intracellular potential, for instance in a finite element model in which the neuron membrane is modeled by a thin-film approximation. We hope that the step-by-step description of these models will make them easy to implement in future studies and will benefit to the design of advanced neural implants and prostheses for the exploration and the rehabilitation of the CNS.

### Conflict of interest statement

The authors declare that the research was conducted in the absence of any commercial or financial relationships that could be construed as a potential conflict of interest.
